# Establishment and validation of nomograms to predict survival probability of advanced malignant pleural mesothelioma based on the SEER database and a Chinese medical institution

**DOI:** 10.3389/fendo.2023.1139222

**Published:** 2023-04-14

**Authors:** Xuemei Zhang, Lele Chang, Yingying Zhu, Yuxin Mao, Tao Zhang, Qian Zhang, Chunbo Wang

**Affiliations:** ^1^ Thoracic Radiotherapy, Harbin Medical University Cancer Hospital, Harbin, China; ^2^ Gastrointestinal Medical Oncology, Harbin Medical University Cancer Hospital, Harbin, China

**Keywords:** advanced malignant pleural mesothelioma, Nomograms, Radiotherapy, SEER database, prognosis

## Abstract

**Objective:**

The purpose of this study was to build nomograms for predicting the survival of individual advanced pleural mesothelioma (MPM) patients using the Surveillance, Epidemiology, and End Results (SEER) database.

**Methods:**

The 1251 patients enrolled from the SEER database were randomized (in a 7:3 ratio) to a training cohort and an internal validation cohort. Eighty patients were enrolled from the Harbin Medical University Cancer Hospital as the external validation cohort. Nomograms were constructed from variables screened by univariate or multivariate Cox regression analyses and evaluated by consistency indices (C-index), calibration plots, and receiver operating characteristic (ROC) curves. Patients from the SEER database who received chemotherapy alone and chemoradiotherapy were statistically paired using propensity score matching of the two groups and performed subgroup analysis in the screened variables.

**Results:**

The nomograms are well-structured and well-validated prognostic maps constructed from four variables: gender, histology, AJCC stage, and treatment. All individuals were allocated into high-risk versus low-risk groups based on the median risk score of the training cohort, with the high-risk group having worse OS and CSS in all three cohorts (*P*<0.05). The outcomes of the subgroup analysis indicated that the advanced MPM patients receiving chemotherapy with or without local radiotherapy do not affect OS or CSS.

**Conclusion:**

The accurate nomograms to predict the survival of patients with advanced MPM were built and validated based on an analysis of the SEER database with an external validation cohort. The study suggests that the additional local radiotherapy to chemotherapy does not increase the survival benefit of patients.

## Introduction

Malignant pleural mesothelioma (MPM), with an international incidence of 1 in 1,000,000, is a rare, aggressive, and often lethal malignancy of pleural origin (1, 2). Survival rates for advanced MPM are very low, with newly published data showing that the median survival for patients with advanced, inoperable is only 12 months (3, 4). Findings from several recent studies have proposed superior efficacy of immunotherapy in patients with MPM, with ipilimumab plus nivolumab extending median survival by 4 months compared with chemotherapy in patients with advanced untreated MPM (5–7). Severe chest pain can occur when the tumor invades the chest wall, and radiotherapy is the preferred treatment for MPM to relieve pain (8).

Nomograms are graphical assessment systems that can quantify risk based on statistical prediction models (9, 10). Many studies have demonstrated that a nomogram can be used as an alternative to the AJCC TNM staging system to individually predict patient prognosis and help physicians to select the best treatment for individual clinicopathological conditions (11–13).

A prognostic model for MPM based on the AJCC I-IV stage has been constructed (14). However, treatment options and survival in advanced MPM differ significantly from those in the early stages. We aimed to build and validate The survival nomograms of patients with advanced inoperable MPM based on the Surveillance, Epidemiology, and End Results (SEER) database.

## Methods

### Data source and participants

Data were obtained from SEER∗Stat (version 8.4.0.1). Patients included in this study must meet (1) the year of diagnosis was between 2005-2018, (2) site code: C34.0-C34.9, C38.4, and ICD- O- 3 histology/behavior codes: 9050-9055 (based on previous literature support) (15), (3) patients who are at stage IIIB and IV. Patients meeting any of the below conditions were excluded (1) patients with a second primary malignancy; (2) patients who survived <30 days or had an unknown cause of death; (3) patients who underwent surgery. All patients were restaged according to the AJCC eighth-edition staging principles. Overall survival (OS) is the time between the date of diagnosis and the date of death from any cause or the last follow-up visit. Tumor-specific survival (CSS) is described as the time between the date of diagnosis and the date of death due to tumor cause or last follow-up. OS and CSS are the primary and secondary endpoints of this study, respectively.

Patients from the SEER database who met the criteria were randomized (in a 7:3 ratio) to a training cohort and an internal validation cohort. The external validation cohort consisted of MPM patients who attended the Harbin Medical University Cancer Hospital from 2010-2018. We used the same screening criteria as those for patients from the SEER database and eventually recruited 80 patients. Follow-up was carried out *via* telephone communication with the patients, and the last follow-up date of November 23, 2022. This a retrospective study based on clinical data, informed patient consent was not required.

### Statistical analysis

SPSS 26.0 (IBM Inc.) and R version 4.1.3 were used for statistical analysis. Patient characteristics were compared between cohorts using a chi-square test. The clinicopathological features significantly related to survival were screened by univariate and multivariate Cox regression analysis, and the variables were further screened using stepwise backward regression, where the model with the smallest Akaike Information Criterion (AIC) score is chosen as the ideal model.

**Figure 1:**
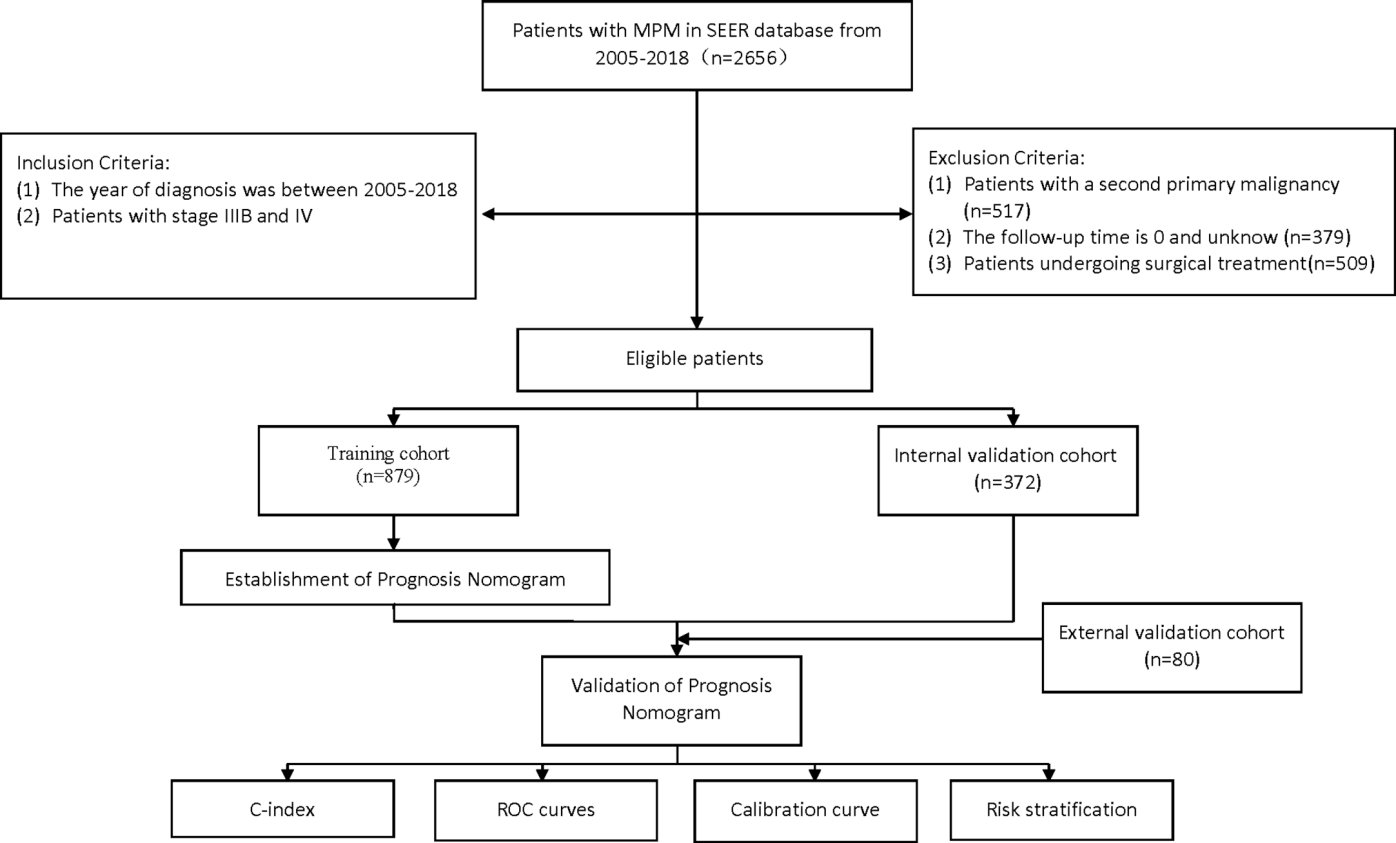
Flowchart of participant inclusion and exclusion.

Finally, nomogram prognostic models that predict the OS and CSS rate of advanced MPM patients at 0.5-, 1-, and 2- years were constructed using the screened variables. We then evaluated the performance in terms of the discriminatory power and accuracy of the models. The discriminatory ability was assessed using the consistency index (C-index) and the area under the receiver operating characteristics (ROC) curve (AUC); calibration curves measure how well the probabilities generated by the nomograms agree with the observed actual probability. A risk score was available for each individual from the nomograms, and the median risk score of the patients in the training cohort was used as the cut-off value to stratify all patients for risk. The Kaplan-Meier survival analysis was conducted to determine if there was a significant difference in OS versus CSS rates in the different risk groups. The flowchart of patient screening and study design is displayed in **Figure 1**.

### Prognostic value of different treatment options in subgroup analysis

Patients from the SEER database who received chemotherapy alone and chemoradiotherapy were statistically paired using propensity score matching of the two groups and performed subgroup analysis in the screened independent risk factors. Cox proportional risk models were used to analyze the relationship between treatment and prognosis in each subgroup. Finally, the results are shown in forest maps.

## Results

### Characteristics of participants

A total of 1251 individuals with MPM were identified in the SEER database and randomized into a training cohort (n = 879) and an internal validation cohort (n = 372), with no differences in clinicopathological and demographic characteristics between the two cohorts.

The 1251 patients from the SEER database were mostly elderly, with more than half of the patients concentrated between 65-85 years of age (67.7%), 974 (77.9%) patients were male, and epithelioid is the most common pathological type (35.6%) in patients with definitive histological diagnosis. AJCC staging showed that most patients were in T4 (64.3%) or N0 (48.4%), and the distribution of patients in stage IIIB and IV were more evenly distributed with 575 (46.0%) and 676 (54.0%) patients, respectively. 49% of patients received chemotherapy alone and 8.3% and 4.7% received chemoradiotherapy or radiotherapy alone, respectively. The external validation cohort from the Harbin Medical University Cancer Hospital had 80 patients, the majority of patients (90.1%) were less than 70 years old, all patients were married Asians, 45% received chemotherapy alone, and 20% received both systemic chemotherapy and radiotherapy to the primary lesion. The clinicopathological characteristics of all patients are outlined in **Table 1**.

**Table 1:** Demographics and clinicopathologic characteristics of the training and external validation cohort.

Characteristics	Training cohort (n = 879) n (%)	Internal validation cohort (n=372) n (%)	Overall(n = 1251) n (%)	External validation cohort(n=80) n(%)	T vs.IV (*P* value)	T vs.EV (*P* value)
Age						
<65	223 (25.4%)	93 (25.0%)	316 (25.3%)	49 (61.3%)	0.57	<0.01
65-75	292 (33.2%)	138 (37.1)	430 (34.4%)	23 (28.8%)		
76-85	305 (34.7%)	117 (31.5%)	422 (33.7%)	7 (8.8%)		
>85	59 (6.7%)	24 (6.5%)	83 (6.7%)	1 (1.3%)		
Gender						
Male	692 (78.7%)	282 (75.8%)	974 (77.9%)	55 (68.8%)	0.24	0.037
Female	187 (21.3%)	90 (24.2%)	277 (22.1%)	25 (31.2%)		
Histology						
NOS	365 (41.5%)	149 (40.1%)	514 (41.1%)	43 (53.8%)	0.82	0.065
Fibrous	146 (16.6%)	61 (16.4%)	207 (16.5%)	10 (12.5%)		
Epithelioid	306 (34.8%)	139 (37.4%)	445 (35.6%)	26 (32.5%)		
Biphasic	62 (7.1%)	23 (6.2%)	85 (6.8%)	1 (1.3%)		
AJCC T						
T1	94 (10.7%)	45 (12.1%)	139 (11.11%)	3 (3.7%)	0.12	<0.01
T2	88 (10.1%)	26 (7.0%)	114 (9.1%)	19 (23.7%)		
T3	57 (6.5%)	14 (3.8%)	71 (5.7%)	22 (27.5%)		
T4	556 (63.3%)	248 (66.7%)	804 (64.3%)	36 (45.0%)		
TX	83 (9.4%)	39 (10.5%)	122 (9.8%)	0 (0.0%)		
AJCC N						
N0	439 (49.9%)	167 (44.9%)	606 (48.4%)	17 (21.3%)	0.15	<0.01
N1	231 (26.3%)	106 (28.5%)	337 (26.9%)	23 (28.7%)		
N2	83 (9.4%)	30 (8.1%)	113 (9.0%)	40 (50.0%)		
NX	126 (14.3%)	69 (18.5%)	195 (15.6%)	0 (0.0%)		
AJCC stage						
IIIB	402 (45.7%)	173 (46.5%)	575 (46.0%)	45 (56.3%)	0.80	0.071
IV	477 (54.3%)	199 (53.5%)	676 (54.0%)	35 (43.7%)		
Race						
White	790 (89.9%)	333 (89.5%)	1123 (89.7%)		0.88	
Black	45 (5.1%)	18 (4.8%)	63 (5.1%)			
Others^a^	44 (5.0%)	21 (5.6%)	65 (5.2%)	80 (100%)		
Grade						
I-II	13 (1.5%)	2 (0.5%)	15 (1.2%)		0.31	
III-IV	75 (8.5%)	30 (8.1%)	105 (8.4%)			
Unknown	791 (90.0%)	340 (91.4)	1131 (94.4%)			
Primary site						
Left	323 (36.8%)	156 (41.9%)	479 (38.3%)	37 (46.2%)	0.23	0.064
Right	519 (59.0%)	202 (54.3%)	721 (57.6%)	43 (53.7%)		
Bilateral	37 (4.2%)	14 (3.8%)	51 (4.1%)			
Marital status						
Married	550 (62.6%)	240 (64.5%)	790 (63.1%)	100 (100%)	0.29	
Single	85 (9.7%)	43 (11.6%)	128 (10.2%)			
Others^b^	244 (27.8)	89 (23.9%)	333 (26.6%)			
Lung metastases						
No	423 (48.1%)	196 (52.7%)	619 (49.5%)	12 (17.3%)	0.30	<0.01
Yes	90 (10.2%)	38 (10.2%)	128 (10.2%)	67 (83.7%)		
Unknown	366 (41.6%)	138 (71.1%)	504 (40.3%)			
Treatment						
Chemoradiotherapy	65 (7.4%)	39 (10.5%)	104 (8.3%)	16 (20.0%)	0.30	<0.01
Chemotherapy alone	437 (49.7%)	176 (47.3%)	613 (49.0%)	36 (45.0%)		
Radiotherapy alone	40 (4.6%)	19 (5.1%)	59 (4.7%)	0 (0.0%)		
No/Unknown	337 (38.3%)	138 (37.1%)	475 (38.0%)	28 (35.0%)		

T, Training cohort; IV, Internal validation cohort; EV, External validation cohort; NOS, not otherwise specified; AJCC Stages, the eighth edition American Joint Committee on Cancer (AJCC) TNM staging system. Others^a^, including Asian or Pacific Islander and American Indian/Alaska Native; Others^b^, including separated, divorced and widowed.

### Screening for independent prognostic factors for model construction

Univariate Cox regression analysis of the training cohort showed age, gender, histology, AJCC stage, and treatment were significantly correlated with survival (*P* < 0.05). The above five variables were subjected to multivariate analysis, and the best model was determined using stepwise backward regression with minimum AIC values. Gender, histology, AJCC staging, and treatment were ultimately identified as independent prognostic factors for modeling the prognosis of advanced MPM. The outcomes of the Cox regression survival analysis based on OS and CSS are presented in **Table 2** and S1, respectively.

**Table 2:** Selection of variables independently associated with OS by univariate and multivariate Cox proportional hazards analysis in the training cohort.

Characteristics	Univariate analysis		Multivariate analysis	
	HR (95% CI)	*P* value	HR (95% CI)	*P* value
Age				
<65	Reference		Reference	
65-75	1.13 (0.94-1.36)	0.19	1.11 (0.93-1.34)	0.24
76-85	1.28 (1.07-1.53)	0.07	1.12 (0.93-1.35)	0.23
>85	1.97 (1.46-2.66)	<0.01	1.44 (1.05-1.97)	0.02
Gender				
Male	Reference		Reference	
Female	0.82 (0.69-0.97)	0.02	0.80 (0.67-0.95)	0.01
Histology				
NOS	Reference		Reference	
Fibrous	1.54 (1.26-1.87)	<0.01	1.64 (1.33-2.01)	<0.01
Epithelioid	0.74 (0.63-0.87)	<0.01	0.80 (0.68-0.94)	<0.01
Biphasic	1.16 (0.87-1.54)	0.31	1.31 (0.99-1.75)	0.08
AJCC T				
T1	Reference			
T2	0.94 (0.7-1.28)	0.71		
T3	0.86 (0.61-1.21)	0.33		
T4	0.89 (0.71-1.12)	0.33		
TX	0.92 (0.68-1.26)	0.62		
AJCC N				
N0	Reference			
N1	1.08 (0.91-1.27)	0.38		
N2	0.91 (0.71-1.17)	0.47		
NX	0.97 (0.79-1.19)	0.80		
AJCC stage				
IIIB	Reference		Reference	
IV	1.23 (1.07-1.41)	0.03	1.37 (1.19-1.57)	<0.01
Race				
White	Reference			
Black	0.88 (0.65-1.20)			
Others^a^	0.91 (0.66-1.25)	0.43		
Grade				
I-II	Reference			
III-IV	1.41 (0.78-2.55)			
Unknown	1.12 (0.65-1.94)	0.25		
Primary site		0.68		
Left	Reference			
Right	1.00 (0.86-1.15)	0.98		
Bilateral	0.74 (0.52-1.06)	0.10		
Marital status				
Married	Reference			
Single	1.17 (0.92-1.48)	0.19		
Others^b^	1.07 (0.92-1.26)	0.37		
Lung metastases				
No	Reference			
Yes	1.21 (0.95-1.53)	0.12		
Unknown	1.00 (0.86-1.15)	1.00		
Treatment				
Chemoradiotherapy	Reference		Reference	
Chemotherapy alone	0.78 (0.60-1.03)	0.08	0.81 (0.62-1.08)	0.15
Radiotherapy alone	1.31 (0.86-1.97)	0.25	1.15 (0.76-1.74)	0.50
No/Unknown	1.38 (1.05-1.83)	0.02	1.42 (1.06-1.89)	0.02

HR, hazard ratio; 95 CI, 95% confidence interval; NOS, not otherwise specified; AJCC Stages, the eighth edition American Joint Committee on Cancer (AJCC) TNM staging system. Others^a^, including Asian or Pacific Islander and American Indian/Alaska Native; Others^b^, including separated, divorced and widowed.

### Creation and validation of nomograms

**Figure 2:**
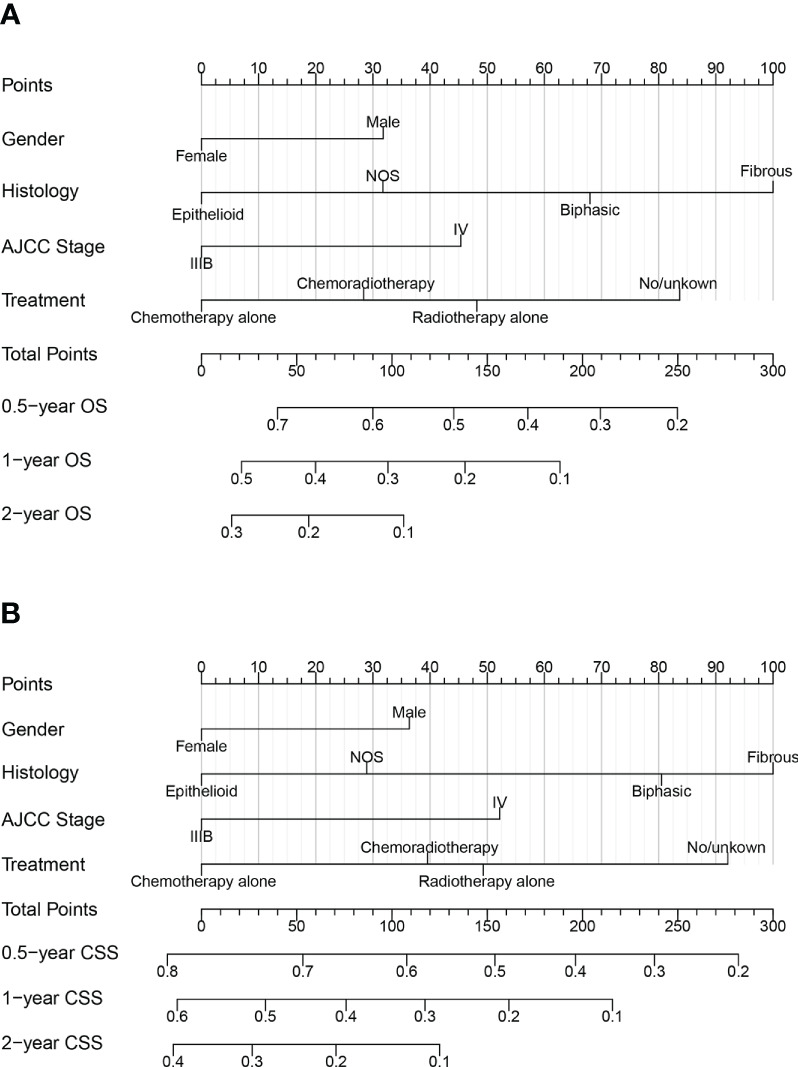
Nomograms for predicting 0.5, 1, and 2year **(A)** OS and **(B)** CSS of patients with advanced MPM.

The nomograms for predicting late survival in MPM patients were established by the four variables screened above (**Figure 2**). By calculating the sum of the scores of the four variables through the nomograms, we could estimate the OS and CSS rate at 0.5-, 1-, and 2- years in patients with advanced MPM. C-index, time-dependent ROC curves, and calibration curves were used to validate the models’ performance. The C-index of the OS-based prediction models for the training, internal validation, and external validation groups were 0.656 (95% CI, 0.636-0.677), 0.668 (95% CI, 0.634-0.701) and 0.684 (95% CI, 0.605-0.763), respectively, as shown in **Table S2**. The CSS-based prediction models had a C-index of 0.654 (95% CI, 0.632-0.675), 0.657 (95% CI, 0.623-0.691), and 0.754 (95% CI, 0.670-0.838), respectively.

**Figure 3:**
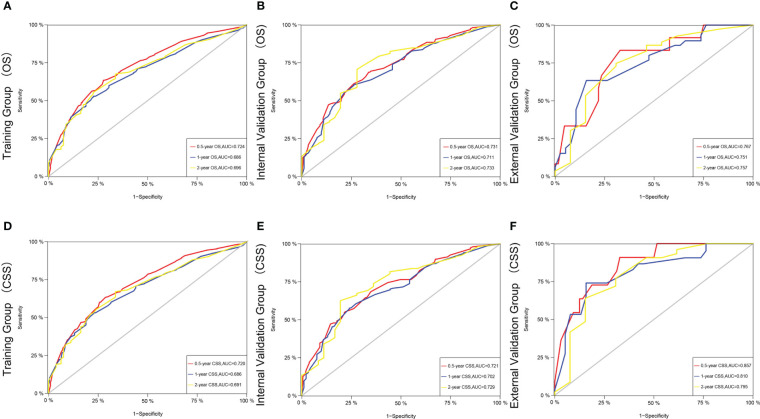
The time-dependent ROC curves of the nomogram predicting OS at **(A)** 0.5-year and 1-year and 2-year in the training cohort, and at **(B)** 0.5-year and 1-year and 2-year in the internal validation cohort, **(C)** 0.5-year and 1-year and 2-year in the external training cohort. The time-dependent ROC curves of the nomogram predicting CSS at **(D)** 0.5-year and 1-year and 2-year in the training cohort, and at **(E)** 0.5-year and 1-year and 2-yearin the internal validation cohort, **(F)** 0.5-year and 1-year and 2-year and 5-year in the external training cohort.

**Figures 3A–C** shows the AUC values of a nomogram predicting 0.5-, 1-, and 2-year OS in three cohorts [training cohort:0.5 year OS 0.724 (95% CI, 0.690-0.758); 1 year OS 0.686 (95% CI, 0.647-0.725); 2 years OS 0.696 (95% CI, 0.641-0.750); internal validation cohort:0.5 year OS 0.731 (95% CI, 0.679-0.784); 1 year OS 0.711 (95% CI, 0.650 -0.771); 2-year OS 0.733 (95% CI, 0.645-0.820); external validation cohort:0.5-year OS 0.767 (95% CI, 0.628-0.905); 1-year OS 0.751 (95% CI, 0.635-0.867); 2-year OS 0.757 (95% CI, 0.596-0.917), respectively]. **Figures 3D–F** demonstrate the AUC values of a nomogram predicting 0.5-, 1-, and 2-year CSS in the three cohorts, respectively.

**Figure 4:**
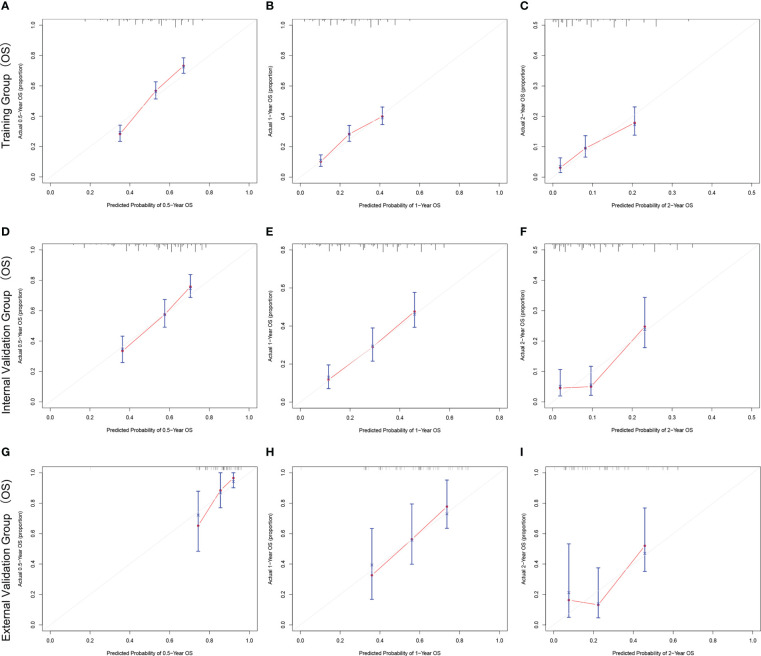
The calibration curves for predicting OS at **(A)** 0.5-year and **(B)** 1-year and **(C)** 2-year in the training cohort, and at **(D)** 0.5-year **(E)** 1-year and **(F)** 2-year in the internal validation cohort, and at **(G)** 0.5-year **(H)** 1-year and **(I)** 2-year in the external validation cohort.

**Figure 5:**
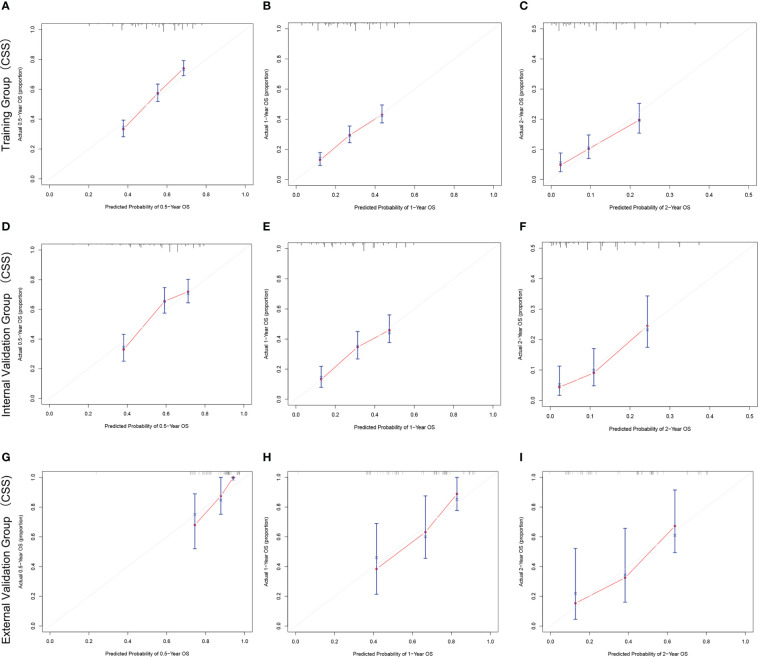
The calibration curves for predicting CSS at **(A)** 0.5-year and **(B)** 1-year and **(C)** 2-year in the training cohort, and at **(D)** 0.5-year **(E)** 1-year and **(F)** 2-year in the internal validation cohort, and at **(G)** 0.5-year **(H)** 1-year and **(I)** 2-year in the external validation cohort.

The C-index and AUC values indicate that the prognostic models have an excellent discriminatory ability for the survival rate of advanced MPM patients. **Figures 4**, **5** show the calibration curves of the prediction model between the actual OS or CSS rates at 0.5-, 1-, and 2- years and the predicted probabilities in the three cohorts, respectively, showing a good consistency between the survival rates generated by the nomograms and the observed survival rates in the actual population.

### Risk stratification based on nomograms

**Figure 6:**
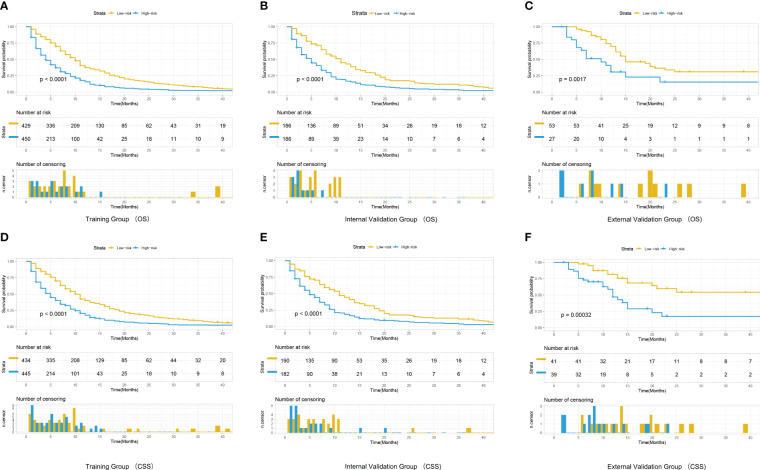
Kaplan-Meier curves for correlation with OS for the low and high- risk groups in the training cohort **(A)**, internal validation cohort **(B)** and external validation cohort **(C)** Kaplan-Meier curves for correlation with CSS for the low and high-risk groups in the training cohort **(D)**, internal validation cohort **(E)** and external validation cohort **(F)**.

A risk score was calculated for all patients by nomograms, and the median risk score in the training cohort (OS: 116, CSS: 128) was used as the cut-off value to allocate patients into a high-risk group (OS: risk score ≥116, CSS: risk score ≥128) and a low-risk group (OS: risk score <116, CSS: risk score <128). Kaplan - Meier survival analysis revealed significant differences in OS or CSS across different risk groups (**Figure 6**), suggesting that nomograms can help us to accurately stratify risk in patients with advanced MPM.

### Prognostic value of local radiotherapy in advanced MPM

**Figure 7:**
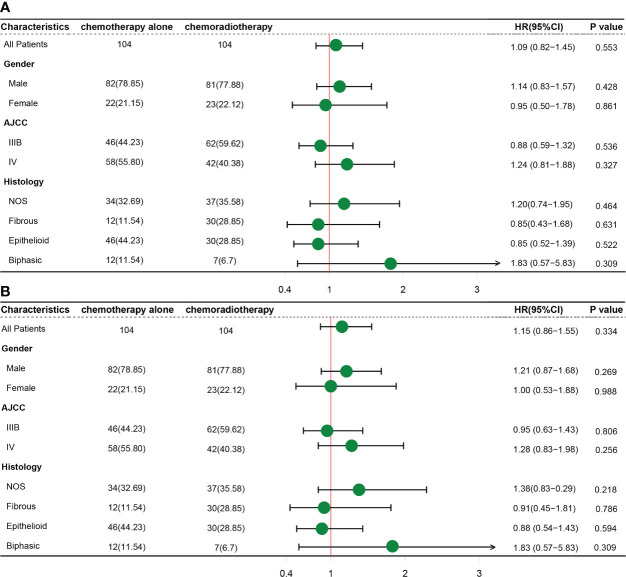
Subgroup analysis of differences in OS **(A)** and CSS **(B)** in the two treatment groups.

Radiotherapy plays a secondary role in patients with MPM compared to the importance of systemic therapy. In addition to systemic treatment, local palliative radiotherapy can relieve pain and improve patients’ symptoms. In our study, SPSS was used for propensity score matching analysis. Pairs of patients receiving chemoradiotherapy or chemotherapy-only were derived using1:1 greedy nearest neighbor matching within PS score of 0.1. This strategy resulted in 104 matched pairs in each group. resulting in 208 patients included in the subgroup analysis. It is evident from the results that the chemotherapy-only group tended to have better OS and CSS in all subgroups, however, all results failed to reach statistically significant (**Figure 7**), indicating that the addition of local radiotherapy to chemotherapy did not provide a survival benefit to patients with advanced MPM.

## Discussion

MPM is a low-incidence rate malignancy, and in previous studies, scholars have had difficulty collecting sufficient case data from a single center. The SEER database is the definitive cancer statistics database in the United States, containing a large sample of U.S. patients from different years, and is convenient and uniquely valuable for studying rare tumors (16–18).

The 1251 patients with advanced MPM from the SEER database in this work were randomized to the training and internal validation cohorts. Univariate analysis indicates that age, gender, histology, AJCC stage, and treatment regimen were significantly related to survival. These five variables were included in a multifactorial Cox regression analysis and further screened for variables using a backward stepwise regression approach. The age variable was excluded from the multifactorial Cox regression, probably because the 2-year survival rate of advanced MPM patients is extremely low and survival status is less affected by age. Nomograms were created based on the other four prognostic variables, then, the C-index, AUC values, and calibration curves validated the discriminatory power and accuracy of the models.

Previous studies have shown a significant difference in the gender distribution of MPM onset and survival rate (19), mainly because the onset of MPM is closely related to asbestos exposure, and those working in asbestos-related industries are mostly male (20). In the present study, the majority of individuals in the three cohorts were male (training cohort: n=692, 78.7%; internal validation cohort: n=282, 75.8%; external validation cohort: n=55, 68.8%) and had worse 2-year survival rates than female (training cohort: 8.9% vs. 16.1%; internal validation cohort. 9.6% vs. 18.6%; external validation cohort: 17.9% vs. 52.7%), further validating gender as an important predictor for survival in MPM patients. Histology significantly affects the survival of patients with advanced MPM, consistent with previous findings that the type of epithelioid was the most common and had a significantly better prognosis than fibrous (14, 21).

Patients with advanced MPM have a single treatment modality, with platinum/pemetrexed chemotherapy being the preferred regimen (3, 22), In recent years, the value of immunotherapy in advanced MPM is also being gradually demonstrated (23). The treatments in this study included: chemotherapy alone, chemoradiotherapy, and radiotherapy alone. Multifactorial analysis revealed that the selection of treatment options was significantly connected to the clinical outcome of MPM patients (*P* < 0.05). Among them, patients who received chemotherapy alone had the best 2-year survival rate (14.1%).

Mesothelioma has long been recognized to be highly radioresistant. Whether radiotherapy is beneficial in MPM patients who have not received extrapleural pneumonectomy has been highly controversial.

Several studies have confirmed that the radiosensitivity of mesothelioma cells is not as low as believed and is even higher than that of non-small cell lung cancer cells (24). Strukowska L et al. found durable local control with palliative radiotherapy targeting primary sites or chest wall metastases at a single dose of ≥4Gy (25).

Ghirardelli et al. performed a retrospective analysis of clinical information on 37 patients with inoperable MPM who progressed locally after first-line chemotherapy, showed that focal radiotherapy (FRT) delayed further systemic therapy in patients (median time of 6 months) and achieved a 1-year regional disease control of 76% in all patients, even better than most reported systemic therapies (26). However, others have suggested that a larger volume of lung irradiated in patients with preserved whole lungs may produce severe pulmonary toxicity and that higher-grade radiation pneumonia is a risk factor in patient mortality (27, 28). Given the persistent lack of high-value clinical evidence on whether local radiotherapy has a survival benefit for advanced MPM, we conducted a subgroup analysis of patients in the well-matched chemotherapy alone versus chemoradiotherapy groups. It was found that there was no remarkable difference in the prognosis with or without radiotherapy in patients who received systemic chemotherapy in advanced stages. Suggesting that radiotherapy may serve as a palliative treatment for local pain relief, but the improvement in symptoms may not translate into a benefit in OS or CSS. However, the patients in the SEER database are mostly from earlier years with outdated radiotherapy techniques. Current sophisticated radiotherapy equipment and precise target volume delineation may improve the benefit of radiotherapy. In addition, the rapid development of proton radiotherapy in recent years may hold great promise for the treatment of superficial malignancies like pleural mesothelioma.

In our study, the median survival of the external validation cohort was better than that of patients from the SEER database (7 months vs. 14 months). Possible reasons for this are (1) the differences in the distribution of clinicopathological factors such as race, marital status, and treatment status among patients in the external validation group compared with those from the SEER database, (2) A relatively large proportion of patients in the external validation group were female compared to the training group, and (3) the smaller number of cases in the external validation group due to the low incidence of MPM and the difficulty of collecting sufficient cases in a single center, which may have led to biased results.

However, the prediction model based on the training cohort was still well validated in the external validation cohort, with remarkable differences in survival among patients in the different risk groups (**Figure 6**). This is the first prognostic model focused on advanced inoperable MPM that differs from previous studies based only on the SEER database, the external validation group in this work demonstrates that the predictive model still has good applicability to patients in real-world Asia. As a retrospective analysis based on a public database, this study has several limitations. First, the SEER database lacks much important clinical information, including specific regimens and timing of treatment for chemotherapy as well as radiotherapy, which are important for the prognosis of MPM patients; second, many variables in the SEER database contain a large number of unknowns (No/Unknow), which may confound the statistical results of the study; third, immunotherapy has been shown to improve the prognosis of advanced MPM patients’ prognosis, however, the SEER database lacks information related to immunotherapy; finally, considering the small sample size in external validation, we only performed a subgroup analysis of patients from the SEER database and lacked validation from real-world data. In summary, real-world multicenter studies are needed to further validate our findings.

## Conclusion

In summary, we successfully constructed and validated two nomograms to predict the survival of advanced MPM patients, providing a more accurate basis for treatment decisions in such patients. Systemic chemotherapy is predominant in patients with advanced MPM, and the addition of radiotherapy failed to improve the probability of survival in advanced MPM.

## Data availability statement

The raw data supporting the conclusions of this article will be made available by the authors, without undue reservation.

## Ethics statement

This is a retrospective study based on clinical data and informed patient consent was not required. The study was conducted with the review and approval of the Board of Directors of the Harbin Medical University Cancer Hospital.

## Author contributions

XZ and LC conceived the original ideas of this manuscript and executed supervision throughout the process. YZ and YM performed the data collection, analysis. TZ and QZ prepared tables, figures and manuscript. CW reviewed and supervised the manuscript. All authors contributed to the article and approved the submitted version.
