# Supplementary material for: Establishment and validation of nomograms to predict survival probability of advanced malignant pleural mesothelioma based on the SEER database and a Chinese medical institution
Source: Front Endocrinol (Lausanne). 2023 Apr 14;14:1139222. doi: 10.3389/fendo.2023.1139222 (PMC10140559; doi:10.3389/fendo.2023.1139222)
Supplement: Supplementary file 1 [file Table_1.docx]

Supplementary Material

**Establishment and validation of nomograms to predict survival probability of advanced malignant pleural mesothelioma based on the SEER database and a Chinese medical institution**

**Xuemei Zhang^1 †^, Lele Chang^2 †^, Yingying Zhu^1†^, Yuxin Mao^1^, Tao Zhang^1^ , Qian Zhang^1^ ,** **Chunbo Wang^1*^**

*** Correspondence:**

Corresponding author's name: Chunbo Wang
Email Address: [13936009550@139.com](mailto:13936009550@139.com)

# Supplementary Tables

Table S1. Selection of variables independently associated with CSS by univariate and multivariate Cox proportional hazards analysis in the training cohort

|  |  |  |  |  |  |  |
| --- | --- | --- | --- | --- | --- | --- |
| Characteristics |  | Univariate analysis | |  | Multivariate analysis | |
|  |  | HR (95% CI) | *P* value |  | HR (95% CI) | *P* value |
| Age |  |  |  |  |  |  |
| ＜65 |  | Reference |  |  | Reference |  |
| 65-75 |  | 1.15 (0.96-1.39) | 0.138 |  | 1.13 (0.93-1.37) | 0.21 |
| 76-85 |  | 1.33 (1.10-1.60) | ＜0.01 |  | 1.16 (0.96-1.41) | 0.13 |
| >85 |  | 1.94 (1.42-2.65) | ＜0.01 |  | 1.42 (1.03-1.97) | 0.03 |
| Gender |  |  |  |  |  |  |
| Male |  | Reference |  |  | Reference |  |
| Female |  | 0.81 (0.68-0.96) | 0.02 |  | 0.80(0.66-0.95) | 0.01 |
| Histology |  |  |  |  |  |  |
| NOS |  | Reference |  |  | Reference |  |
| Fibrous |  | 1.50 (1.22-1.85) | ＜0.01 |  | 1.60（1.29-1.98） | ＜0.01 |
| Epithelioid |  | 0.77 (0.65-0.90) | 0.01 |  | 0.83（0.71-0.98） | 0.03 |
| Biphasic |  | 1.26 (0.94-1.67) | 0.12 |  | 1.42（1.06-1.89) | 0.02 |
| AJCC T |  |  |  |  |  |  |
| T1 |  | Reference |  |  |  |  |
| T2 |  | 0.95 (0.69-1.30) | 0.73 |  |  |  |
| T3 |  | 0.89 (0.62-1.27) | 0.51 |  |  |  |
| T4 |  | 0.90 (0.71-1.14) | 0.39 |  |  |  |
| TX |  | 0.94 (0.68-1.30) | 0.71 |  |  |  |
| AJCC N |  |  |  |  |  |  |
| N0 |  | Reference |  |  |  |  |
| N1 |  | 1.05 (0.89-1.25) | 0.54 |  |  |  |
| N2 |  | 0.91 (0.70-1.17) | 0.45 |  |  |  |
| NX |  | 0.94 (0.76-1.16) | 0.57 |  |  |  |
| AJCC stage |  |  |  |  |  |  |
| IIIB |  | Reference |  |  | Reference |  |
| IV |  | 1.26 (1.09-1.45) | ＜0.01 |  | 1.39(1.20-1.61) | ＜0.01 |
| Race |  |  |  |  |  |  |
| White |  | Reference |  |  |  |  |
| Black |  | 0.80 (0.57-1.11) | 0.18 |  |  |  |
| Others^a^ |  | 0.81 (0.58-1.14) | 0.23 |  |  |  |
| Grade |  |  |  |  |  |  |
| I-II |  | Reference |  |  |  |  |
| III-IV |  | 1.37 (0.74-2.53) | 0.32 |  |  |  |
| Unknown |  | 1.15 (0.65-2.04) | 0.63 |  |  |  |
| Primary site |  |  |  |  |  |  |
| Left |  | Reference |  |  |  |  |
| Right |  | 1.00 (0.86-1.16) | 0.99 |  |  |  |
| Bilateral |  | 0.75 (0.52-1.09) | 0.13 |  |  |  |
| Marital status |  |  |  |  |  |  |
| Married |  | Reference |  |  |  |  |
| Single |  | 1.18 (0.92-1.51) | 0.18 |  |  |  |
| Others^b^ |  | 1.09 (0.93-1.28) | 0.28 |  |  |  |
| Lung metastases |  |  |  |  |  |  |
| No |  | Reference |  |  |  |  |
| Yes |  | 1.22 (0.96-1.56) | 0.11 |  |  |  |
| Unknown |  | 0.97 (0.83-1.12) | 0.65 |  |  |  |
| Treatment |  |  |  |  |  |  |
| Chemoradiotherapy |  | Reference |  |  | Reference |  |
| Chemotherapy alone |  | 0.75 (0.57-0.99) | 0.04 |  | 0.77(0.58-1.02) | 0.07 |
| Radiotherapy alone |  | 1.19 (0.78-1.83) | 0.43 |  | 1.06(0.69-1.63) | 0.8 |
| No/Unknown |  | 1.32 (1.00-1.75) | 0.05 |  | 1.34(1.00-1.80) | 0.04 |
|  |  |  |  |  |  |  |

HR, hazard ratio; 95 CI, 95% confidence interval; NOS, not otherwise specified; AJCC Stages, the eighth edition American Joint Committee on Cancer (AJCC) TNM staging system. Others^a^, including Asian or Pacific Islander and American Indian/Alaska Native; Others^b^, including separated, divorced and widowed.

| Table S2. C-index for the nomograms and TNM 8th stage in patients with advanced MPM | | | | | | |
| --- | --- | --- | --- | --- | --- | --- |
|  | Training set | | Internal validation set | | External validation set | |
|  | OS (95%Cl) | CSS (95%Cl) | OS (95%Cl) | CSS (95%Cl) | OS (95%Cl) | CSS (95%Cl) |
| Nomogram | 0.656(0.636-0.677) | 0.654 (0.632-0.675) | 0.668 (0.634-0.701) | 0.657 (0.623-0.691) | 0.684 (0.605-0.763) | 0.754 (0.670-0.838) |
| TNM 8th stage | 0.544 (0.523-0.565) | 0.548 (0.527-0.569) | 0.568 (0.537-0.599) | 0.569 (0.537-0.601) | 0.573 (0.502-0.645) | 0.586 (0.502-0.670) |
